# Understanding and applying pharmacometric modelling and simulation in clinical practice and research

**DOI:** 10.1111/bcp.13119

**Published:** 2016-09-29

**Authors:** Joseph F. Standing

**Affiliations:** ^1^ Infection, Immunity, Inflammation SectionUCL Institute of Child Health30 Guilford StreetLondonWC1N 1EH; ^2^ Department of PharmacyGreat Ormond Street Hospital for ChildrenLondonWC1N 3JH; ^3^ Paediatric Infectious Diseases Research GroupSt George's, University of LondonCranmer TerraceLondonSW17 0RE

**Keywords:** pharmacodynamics, pharmacokinetics, pharmacokinetic–pharmacodynamic modelling

## Abstract

Understanding the dose–concentration–effect relationship is a fundamental component of clinical pharmacology. Interpreting data arising from observations of this relationship requires the use of mathematical models; i.e. pharmacokinetic (PK) models to describe the relationship between dose and concentration and pharmacodynamic (PD) models describing the relationship between concentration and effect. Drug development requires several iterations of pharmacometric model‐informed learning and confirming. This includes modelling to understand the dose–response in preclinical studies, deriving a safe dose for first‐in‐man, and the overall analysis of Phase I/II data to optimise the dose for safety and efficacy in Phase III pivotal trials. However, drug development is not the boundary at which PKPD understanding and application stops. PKPD concepts will be useful to anyone involved in the prescribing and administration of medicines for purposes such as determining off‐label dosing in special populations, individualising dosing based on a measured biomarker (personalised medicine) and in determining whether lack of efficacy or unexpected toxicity maybe solved by adjusting the dose rather than the drug. In clinical investigator‐led study design, PKPD can be used to ensure the optimal dose is used, and crucially to define the expected effect size, thereby ensuring power calculations are based on sound prior information. In the clinical setting the most likely people to hold sufficient expertise to advise on PKPD matters will be the pharmacists and clinical pharmacologists. This paper reviews fundamental PKPD principles and provides some real‐world examples of PKPD use in clinical practice and applied clinical research.

## **Introduction**

When a medicine is prescribed, the purpose is to derive an effect that usually evolves with time. Whilst the definition of a medicine now encompasses small molecules, biologics and gene therapy, there remains a fundamental requirement to understand the dose–response relationship in order to determine how much and how frequently to administer a treatment. Paracelsus, widely regarded as the founder of modern toxicology, wrote: “Poison is in everything… the dosage makes it either a poison or a remedy”. This should be considered regardless of the treatment in question: for example, marathon runners often poison themselves by drinking too much water 1. It is important to consider dosing in both clinical practice and research, which requires an understanding of pharmacokinetics (PK) and pharmacodynamics (PD).

In this paper the term modelling refers to mathematical and statistical modelling. The mathematical model is defined as an equation used to relate known covariates (dose given, time of dose, time of observations) with observed measurements. In the case of the PK 1 compartment intravenous bolus model, the concentration measured at time *t* is given by: ĉ(*t*) = *D*/*Ve* ^*–CL/Vt*^, where *D* is the known dose, *V* and *CL* are the model parameters volume of distribution and clearance, and ĉ(*t*) is the model prediction of concentration at some time *t*. Using this assumed mathematical model, the values of the parameters *V* and *CL* are sought that minimise the difference between the model prediction and observed concentrations; this is the statistical model, which in the simplest case of single subject data, is given by: *c*(*t*) = ĉ(*t*) + ε, where *c*(*t*) is an observed concentration at time *t*, and ε denotes the deviation of the model prediction from the observation. Statistical modelling seeks to minimise the value of ε by searching for the optimal values of the model parameters (*V* and *CL*). All models are gross simplifications of the system under study, and so the goal of PKPD modelling is often to test a range of models to determine which fits best. Clinical pharmacologists tend to choose specific models using a knowledge of the system that generated the observed data; this will be discussed in more detail below.

In clinical practice and research, the study of PK only is usually confined to a limited set of circumstances where the PD can be readily inferred from a measured concentration. An example of this is antimicrobial chemotherapy, where the relationship between the minimal inhibitory concentration (determined *in vitro*) is linked with maximum concentration (*C*_*max*_), area under the (concentration–time) curve (AUC) or fraction of a dose interval is spent with concentrations above the minimal inhibitory concentration 2. In many situations one wishes to also model the PD in order to understand the full dose–concentration–effect relationship, or occasionally one may not have easy access to PK measures and seek to model the dose–response, otherwise known as K‐PD models. PD can encompass a wide variety of measurement types, all of which can be described in mathematical terms with parameter values estimated using statistical modelling. In recent years, the term pharmacometrics has gained popularity. Pharmacometrics encompasses the analysis of PK and PD data, and then uses resulting models to make inferences (often using simulation) on optimum dosing for clinical trials or practice.

An understanding of pharmacometric modelling and simulation, and how it can give insights into the dose–concentration–effect relationship, will be useful to all clinical pharmacists and pharmacologists. This article seeks to set out the basic principles, firstly through providing detailed answers to a series of common questions, followed by a section giving some brief examples on some real‐world applications of pharmacometric modelling in clinical practice and research.

## **Why are (mechanism‐based) mathematical and statistical models required to understand pharmacometric data?**

**Figure 1:**
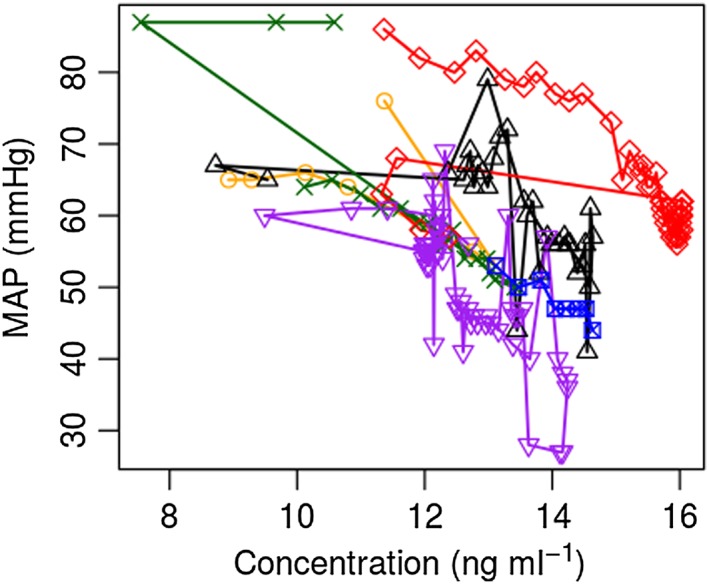
Model predicted remifenatanil concentration *vs.* mean arterial pressure (MAP) in infants prior to craniofacial surgery 3. Different symbols and colours represent data points from each patient

Biological systems are inherently nonlinear, and defining a target exposure or concentration through simple observations of raw data can be difficult. For example, in Figure 1, a plot of the PK model‐predicted remifentanil concentrations *vs.* observed mean arterial blood pressure (MAP) measurements have been made using data collected on a study in infants prior to craniofacial surgery 3. The anaesthetists in this study used remifentanil to control MAP in order to reduce bleeding in the operative field. The aim of this study was to therefore combine measurement of remifentanil PK with measures of MAP (PD) to estimate the parameters of a PKPD model that would be used to define a target concentration (along with appropriate dose to reach that concentration) to yield a 30% drop in MAP. Through simple observation of these data, defining an appropriate target concentration is challenging for two main reasons:

Firstly, hysteresis is clearly present in that the same effect (MAP) can be seen at different observed concentrations within a patient. This comes about due to the fact that circulating concentrations are in flux coupled to the delay in the drug reaching the site of action, binding to its target and eliciting its effect. Nonlinear mathematical PK and PD models coupled with an effect compartment model were used to describe this phenomenon, define the target effect site concentration, and then to suggest a dose yielding this concentration in a typical patient. Here the word *nonlinear* refers to the fact that the PK (a two‐compartment model) and the PD (sigmoidal *E*_*max*_ model) were not expressed as linear *y* = *mx* + *c* type models. The term *effect compartment* refers to an additional compartment with first order equilibration rate constant between it and the central compartment, which was used in the PD model to account for hysteresis.

The concept of a typical patient, or average expected response in the population of interest, brings us to the second challenge for interpreting these data: namely that there is a clear interindividual variability between patients. Ignoring the correlation between each individual's data points when fitting the PKPD model (the so‐called naïve pooled approach) may bias parameter estimates and will inflate the amount of unexplained variability in the model. For this reason, mixed effects analysis, or the so‐called *population approach*, must be used for parameter estimation during statistical model fitting 4, 5, 6, 7, 8. For a full account of the model‐building process in this example, readers are referred to the original article, which contains an appendix of the model code 3.

## **What *biological prior* information do pharmacometric modellers use to inform model choices?**

Beware the mathematician or statistician who, upon seeing PK or PD data, questions the proposed pharmacological model and suggests an empirical alternative. At its extreme, statisticians are now suggesting multimodel approaches whereby several models are simultaneously fitted, the weight given to each model adjusted according to how well it fits the data 9. Whilst such approaches are undoubtedly useful for fitting and describing observed data, large sample sizes and exposure ranges will be required to characterise the population response and extrapolation outside the studied population will not be straight‐forward without biologically interpretable parameters. By ignoring the extensive biological prior information that we, as pharmacologists, have on the system that generated the data, empirical modelling approaches are rarely useful for application in clinical settings where small datasets are available, and the goal is often to extrapolate findings in one population to another, to use findings of one study to plan another (the learning and confirming paradigm 10), or to apply findings to dose adjustment in direct patient care.

In the case of a physiologically‐based PK (PBPK) model, with tissue volumes, blood flows and partition coefficients added to the model *a priori* rather than fitted to observed PK data, it is clear from where the biological priors come. However, even the simple 1‐compartment PK model 11 parametrised with clearance (CL) and volume (V) carries biological interpretation.

The fact that CL is a parameter with units of volume per time, which match blood flows and glomerular filtration rates (GFRs) for example, and is related to the AUC through CL = dose/AUC allows one to leverage prior information. For example, the CL of tobramycin, which is eliminated primarily by glomerular filtration, in a typical 70 kg individual is around 140 ml min^−1^ 12, or slightly higher than a normal GFR. Say tobramycin was a new drug, and from its physicochemical properties (large polar molecule) and preclinical data (excreted unchanged in the urine) one knew it was likely to be excreted by glomerular filtration, first‐in‐man dosing could be rationally planned to attain target concentrations (recall that average concentration is given by *AUC*_(0‐t)_/*t*) in the desired nontoxic ranges, and it would come as no surprise to the pharmacologist to find CL to be similar to GFR in these studies. This biological prior knowledge would then allow dosing to be planned to attain target concentrations in subsequent studies in populations with different GFRs (e.g. elderly, hyperfiltrating ICU patients, children).

In the case of V, the apparent volume of distribution, whilst it does not represent the volume of an actual physiological compartment, biological prior information can still be utilised. For example, diclofenac is highly bound to plasma proteins, so it should come as no surprise that its estimated central V for a typical 70 kg individual is around 3.68 l 8, which is similar to normal plasma volume 13. For a highly protein bound drug such as diclofenac, one would expect V to have a linear relationship with body weight since blood volume is proportional to weight 13. For drugs with larger distribution volumes, particularly where partition to body fat maybe important in obese patients, models for predicting lean body weight or fat‐free mass are now available for adults 14, 15 and children 16. Using biological prior information such as this can help to predict maximal concentrations and elimination half‐lives in populations of interest.

With regard to PD models, there are two main considerations. The first is on the observed response, its time course and its type (usually a measured biomarker or clinical outcome such as disease score). The observed response will be heterogeneous and disease specific and whether biological prior information can be used to inform modelling is variable. For example, drug‐induced neutropaenia has been successfully described using a mechanistic model of the simplified life‐span of a neutrophil, with most cytotoxic chemotherapy agents acting on the proliferating precursor cells 17. On the other hand, sometimes PD endpoints are measured by a score (for example the Paediatric Crohn's Disease Activity Index 18) whereby the introduction of biological prior information on probabilistic PD models is less straightforward. The second consideration with PD models is the concentration–response effect at the site of drug action, which is then often used to drive the observed PD time course, often through an effect compartment, or using indirect response models 19. Here, the well‐established Hill (or Emax) model is used, which can be derived from the law of mass action (see derivation in 20), and provides a mechanistic basis for the concentration–effect relationship.

## **How do pharmacometric models scale with size and age?**

In paediatrics, it is well established that smaller children need smaller doses, but this is often lost in the one‐dose‐fits all world of adult medicine. Adult clinical studies in high‐profile journals often do not recognise that body size is an important determinant of drug exposure and consequently ought to be corrected for. This is particularly important for drugs requiring optimised exposure for effect (e.g. anti‐infective agents) or those with a narrow therapeutic index. For example, Nijland et al 21 state that rifampicin exposure is *strongly reduced* in patients with type II diabetes whereas the majority of the difference in exposure between nondiabetics and Type II diabetics is explained by the Type II diabetics being heavier. Takahashi et al 22 meanwhile emphasised genetic differences as the major causative factor behind an observation that a cohort of African–American, Caucasian and Japanese patients needed different doses of warfarin. Body weights in these cohorts were not matched, and the African–American patients were heavier than the Caucasian patients, who in turn were heavier than the Japanese patients. Dividing the dose by the weight shows all ethnic groups in this study were on 0.06 mg kg^−1^, and from the reported regression coefficients being heterozygous for any CYP2C9 polymorphism or VKORC1 1173 C > T has the same effect as a 55 kg or 42 kg difference in body weight, respectively. This poses the question as to why a flat 10 mg induction regimen is recommended in all adults whether they weigh 40 kg or 120 kg, whereas prescribers are warned about potential genotypic effects in the summary of product characteristics.

Accepting that PK scales with size, it is then important to consider how important PK parameters scale. In 1947, in his treatise on scaling of basal metabolic rate with size and it implications, Kleiber 23 stated:

“For the dosage of drugs one should know whether or not the action depends on reaching a certain concentration in the blood stream without regard to its further maintenance. In this case the dosage should be proportional to body weight, since the amount of blood is proportional to body weight. If, however, the action of the biotic depends on the maintenance of a given concentration over a period of time, and if the rate of destruction or excretion of the biotic is proportional to the metabolic rate, then the dosage should be based on the metabolic body size.”

In other words, volumes scale with linear body weight, and hence drugs for which a threshold concentration is required (e.g. aminoglycoside *C*_*max*_ targets) should be dosed by body weight, whereas, in the case of most drugs, where PD is driven by exposure (recall *AUC* = *dose*/*CL*) metabolic weight, meaning weight raised to a power of 3/4, should be used. This metabolic weight is quite similar to body surface area (weight raised to a power of 2/3) and paediatricians have long since known to dose narrow therapeutic index drugs by surface area 24. Scaling of clearance by metabolic weight does not only apply to small molecules, but also biologics 25. Here it must be noted that these principles apply to drugs with both linear and nonlinear pharmacokinetics, but with Michaelis–Menten elimination it is Vm that should scale allometrically, with Km constant across size and ages.

**Figure 2:**
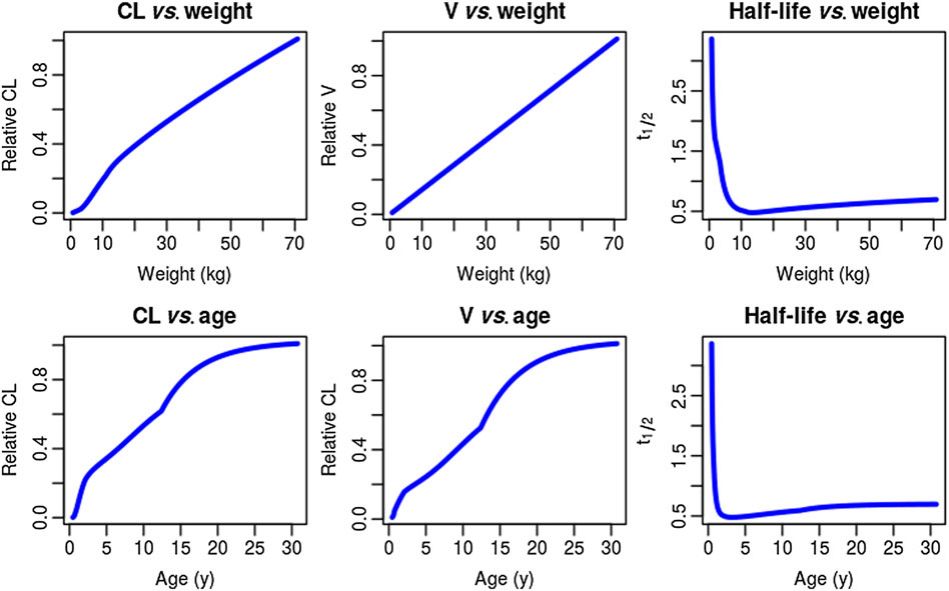
Illustration of the relative expected changes in clearance (CL) volume of distribution (V) and elimination half‐life with weight (based on allometric principles) and age for the one‐compartment intravenous bolus model. The standard value for weight here is set to 70 kg, so CL and V take the value 1

Returning to the linear PK case, this difference in the way that CL and V scale with size means that smaller people have shorter half‐lives due to their proportionally higher CL and therefore elimination rate constant. Figure 2 gives an illustration of how CL, V and half‐life are expected to vary with size and age according to the principles described above. Unfortunately, this subtlety was lost in a *British Medical Journal* study finding shorter caffeine half‐lives (the paper incorrectly asserts half‐life to be a proxy for CL) in pregnant women were associated with lower birth weights (*P* = 0.06), caffeine with its metabolites being somehow responsible for fetal growth restriction 26. There are three fundamental problems with the analysis of these data that would be spotted by a competent PK expert. Firstly, one cannot estimate half‐life from oral PK data without also estimating an absorption rate constant (which might reasonably be fixed to a sensible value if no data in the absorption phase were gathered), V (to transform the dose input into a concentration) and CL (from which and V, the elimination rate constant can be derived along with half‐life should one wish). The authors do not mention V or absorption rate constant, so immediately the reader will be concerned about how exactly half‐life was estimated. The second problem is that empirical linear covariate analysis was done on half‐life, including correlated items such as age and weight, without prior consideration of the biological system that derived the data. Caffeine is largely eliminated by CYP1A2‐mediated hepatic metabolism 27 so one would expect larger individuals with larger livers to have a greater capacity for caffeine metabolism. Given the weight range reported, the caffeine half‐life for the smallest woman would be approximately 20% shorter than that of the largest woman based on allometric principles, and this relationship would be nonlinear. Given that we therefore expect smaller women to have shorter half‐lives, and given that birth weight and maternal weight are correlated so we expect smaller women to have smaller babies, ideally one would correct for size *a priori* to delineate this effect from other covariates of interest. Finally, half‐life is a continuous variable yet the authors arbitrarily dichotomised the group into *fast* and *slow* finding a weak (*P* = 0.06) association between *fast* half‐life and low birth weight. It would have been interesting to see whether this relationship would have held had CL been estimated and the test conducted on this continuous variable.

Size scaling, when done properly as described above, tends to work well in children older than 2 years but, in younger patients, maturation of drug metabolising enzyme expression and glomerular filtration means that age needs to be taken into account. Various methods for size and age scaling are available, the most sensible of which was recently set out and proposed as a standard scaling method 28. Whilst size and age scaling are now well established for PK models, and it has been proposed in many cases that with proper PK scaling PD can be predicted in children 29, there are some PD endpoints (e.g. drug effects on the developing adaptive immune system 30) where size and age may need to be considered in PD modelling.

## **What is meant by *population* PKPD?**

The population approach when applied in pharmacometrics is used to refer to the statistical aspect of model fitting called mixed effects or multilevel modelling. By fitting models, the goal is to estimate the most likely values of parameters (e.g. CL and V) from a set of observed data (e.g. concentrations) along with known covariates (e.g. dose, time, body weight). Whilst the standard goal of model fitting by regression is to minimise a residual departure of model predictions from observed data points, since PKPD data include multiple data points from several individuals, it is necessary to account for the correlations of data points within individuals so as not to bias parameter estimates and inflate unexplained variability. For this reason, in addition to a data point level of variability, mixed effects modelling has a parameter level of variability, allowing parameters to vary between individuals. Population analysis can be used for rich or sparse data. The rule‐of‐thumb definition for rich data is at least as many samples per subject as model parameters 31, with fewer samples per subject than model parameters being called sparse data. This definition may, however, be too simplistic because the timing of samples can be more important than the total number. For example, multiple samples towards the end of an oral administration PK curve will give little information on volume and absorption parameters. It is now possible to use optimal design, a technique using a previous or assumed model, to define optimally informative sampling times so that data collected will give precise estimates of model parameters 32. The end result of a population pharmacometric model fitting exercise is a set of typical population model parameters, their variance, and the variance of the residual unexplained variability. This can then be used to generate new hypotheses (e.g. what will be the expected concentrations under different dosing conditions) or as a Bayesian prior for personalised medicine. There are several excellent reviews explaining the population approach to PKPD analysis 5, 7, 33.

## **Some examples of pharmacometric models used in the clinic**

### Dosing in special populations

Prescribing medicines outside the terms of their product license (off‐label) is common in hospital‐based settings, particularly in paediatrics. Often when this is the case it may be appropriate to adjust dosing and here the clinical pharmacist or pharmacologist with a sound understanding of PKPD principles will be able to help. An example of this arose in around 2009 when the infectious diseases team at Great Ormond Street Hospital wanted to use posaconazole, which was unlicensed in children. At the time, there were published PK data on only two children (aged 8 and 10 years) 34 so to predict dosing in younger patients required extrapolation. It was known that posaconazole is hepatically metabolised (so CL should follow allometric 3/4 scaling) and that the major metabolic pathway was glucuronidation. The maturation of glucuronidation was known through studies on paracetamol and morphine 35. Assuming that the target exposure should be achieved by a dose achieving a similar AUC to the licensed adult dose (200 or 400 mg), simply multiplying this dose by a scaled typical weight for age and additionally the published maturation function 35 (which takes values between 0 and 1, increasing with age) gave the following target doses to give a similar exposure to 200 mg in adults: neonates 1.5 mg kg^−1^; infants aged 1 month‐1 year 3 mg kg^−1^; infants and children aged 1 year or over 4 mg kg^−1^ (maximum 200 mg). These doses were then put forward to be used in the hospital prescribing guidelines.

### Personalised medicine

The term personalised medicine, which in recent years has been hijacked by reductionist (pharmaco‐)geneticists, can have two meanings. Firstly, there is stratified medicine, whereby treatment is personalised before a dose is given. Sometimes this might be a genetic or other clinical/biomarker that determines treatment choice, sometimes this might be a clinical/biomarker that determines treatment dose. Paediatricians practice this second type of stratified medicine every time a drug is prescribed, the dosing being based on weight, age or surface area. Population PKPD models can be used to identify important covariates that determine response, and this may include genetic or metabolomic markers alongside other factors, ideally informed by biological prior information.

The second interpretation of personalised medicine arises where a treatment is adjusted according to response (this may also be called individualised medicine). Typically, this is done with therapeutic drug monitoring, where the biomarker is drug concentration, although the biomarker can just as easily be a PD endpoint (e.g. International Normalized Ratio in response to warfarin). In this context, rather than empirically adjusting the dose until a target is reached, there is increasing interest in using population PKPD models as Bayesian priors, with the observed patient biomarker and covariate information being used to construct a posterior set of most likely individual model parameters to be used to predict/adjust future treatment. A wide range of software applications are now available for this 36, and applications beyond traditional therapeutic drug monitoring 37 such as prediction of drug‐induced neutropaenia 38 and International Normalized Ratio under warfarin therapy 39.

### Managing overdose

PK principles are crucial in some overdose situations, most notably with paracetamol where a PK‐derived nomogram is available to guide acetylcysteine therapy based on measured paracetamol concentration. The PKPD‐literate clinical pharmacist or pharmacologist does not, however, need to limit advice giving solely to agents that have readily available nomograms. One example was a query received regarding persistent hypotension in a child following an accidental intravenous clonidine overdose. Using a published PKPD model linking clonidine and mean arterial pressure (MAP) 40, the trajectory of the expected hypotensive time course given the dose received was easily plotted to show that resolution of the effect would be expected to take several more hours.

## **Examples of pharmacometric models in clinical pharmacology research**

### Developing dosing guidelines for a clinical trial

Pivotal clinical trials are often costly and time consuming so getting the dose right, particularly for narrow therapeutic index agents, is of critical importance. Whilst many pharmaceutical companies integrate PKPD information throughout early‐phase development to get the dose right for Phase III, it is also important that investigator‐led clinical studies are supported by clinical pharmacist or pharmacologist colleagues in study design. An example of this was during the design of a study to use insulin‐like growth factor (IGF‐1) in children with Crohn's disease 18. It was proposed that IGF‐1 supplementation could be used to promote growth in adolescents with Crohn's disease and IGF‐1 deficiency, but since high IGF‐1 levels may be carcinogenic, it was important to ensure that the dosing would only correct levels up to the normal range and not too far beyond. A small PK study was performed in the target population, and covariate analysis showed that in addition to the biological priors of size and age (normal IGF‐1 levels increase during adolescent growth), disease severity measured by the PCDAI was required to tailor dosing to endogenous IGF‐1 production.

### Defining the effect size for a clinical trial

The number of patients recruited to a clinical trial is governed by the expected effect size of the tested treatment. In many cases, investigators make over‐optimistic predictions on the expected effect size leading to trail failure due to inadequate sample size 41. This is an area where PKPD model predicted outcomes are underutilised. In the planning of a randomised double blind noninferiority trial of clonidine *vs.* midazolam for sedation in neonatal and paediatric intensive care, the clinician's estimate of successful sedation with midazolam (the control arm) was 85%, which led to a sample size of 90 (45 patients per group). The midazolam effect size was also evaluated through simulation of expected concentrations using the planned dose scheme, a published PK model 42, 43 and a target PD concentration 44. This gave an expected sedation success rate of 75%, which meant the necessary sample size increased more than three‐fold to 300 patients (150 per group), and indeed this more conservative effect size will be used in the proposed study.

### Pharmacometric model parameters as trial endpoints

In the drive to make clinical trials more efficient, an interesting idea has recently been proposed whereby the drug effect parameter in a PKPD model could be used as a trial endpoint. Often trial endpoints are tested as being a biomarker or clinical observation at a specific, somewhat arbitrary time, whereas if a drug effect parameter were used, data from the whole response time course within a patient could be used. Using the example of a viral kinetic model in hepatitis C infection, Laouenan et al. 45 showed that the power to detect a difference in drug effect between two competing therapies could in some circumstances increase study power by 10‐fold as compared with using the single time‐point outcome of change in viral load at Day 14 of treatment. For diseases where there are sufficient data to build a prior population PKPD model, this approach could be a paradigm for small investigator‐led clinical trial design.

## **Conclusion**

In this paper, arguments for ensuring that pharmacometric knowledge remains a central pillar of clinical pharmacology have been given. The implications of scaling PKPD responses (in particular for size) and of interindividual variability in responses meaning individualisation of dosing or treatment choice maybe required, are not always appreciated by the wider clinical research community. Clinical pharmacists and pharmacologists with this knowledge are ideally placed to influence clinical management using pharmacometric principles. In addition, clinical trial design is increasingly being seen as a specialist statistical subject. Clinical trials methodologists may not fully appreciate the value of the biological prior information won over decades of clinical pharmacology research; there seems a clear need for clinical pharmacists and pharmacologists to become more involved in clinical trial design.

## **Competing Interests**

The author has completed the Unified Competing Interest form at www.icmje.org/coi_disclosure.pdf (available on request from the corresponding author) and declares: no support from any organisation for the submitted work; no financial relationships with any organisations that might have an interest in the submitted work in the previous 3 years; no other relationships or activities that could appear to have influenced the submitted work.

*J.F.S. received funding from a* *United Kingdom Medical Research Council Fellowship* *(grant* *G1002305* *)*.

## **Contributors**

J.F.S. wrote the manuscript based on a teaching session given at the 2015 BPS Winter Meeting.
